# Basic reproductive biology of daggertooth pike conger, *Muraenesox cinereus*: A possible model for oogenesis in Anguilliformes

**DOI:** 10.1186/s40851-015-0025-0

**Published:** 2015-09-03

**Authors:** Yasuhisa Kobayashi, Tsuyoshi Mototani, Fumiyasu Murayama, Tatuya Sakamoto

**Affiliations:** Ushimado Marine Institute (UMI), Faculty of Science, Okayama University, Ushimado, Setouchi, 701-4303 Japan; Okayama Prefectural Technology Center for Agriculture, Forestry and Fisheries, Research Institute for Fisheries Science, Ushimado, Setouchi, 701-4303 Japan

**Keywords:** Conger, Eel, Gonad, Ovary, Testis, Reproduction

## Abstract

**Introduction:**

Eels are animals commonly used in zoological research, as these species have a unique catadromous life history and belong to a phylogenetically ancient group of Teleostei. However, eel reproduction is difficult to investigate, since mature samples are not easily obtainable in the wild. In this study, we tested daggertooth pike conger (*Muraenesox cinereus*), an Anguilliformes species, as a potential model for the investigation of the reproductive biology of eels. Seventy individuals were caught between June and October, which is supposed to be their spawning season, from inshore of the Seto Inland Sea.

**Results:**

The lengths and ages of samples ranged from 510 to 1239 mm and three to nine years, respectively, and the sex ratio was skewed towards females (96 % of the total sample). The gonado-somatic index of the females peaked in July. Histological observation revealed that these ovaries were similar to those of other eel species and contained matured oocytes (migratory-nucleus stage), suggesting that pike conger spawn inshore in July. The plasma concentrations of sex steroid hormones (estradiol-17β and 11-keto-testosterone) in females gradually increased during maturation and decreased after spawning, indicating the involvement of these hormones in oogenesis of pike conger.

**Conclusions:**

The present study is the first to report on characteristics of natural oogenesis in pike conger. Because naturally maturing samples can easily be captured, daggertooth pike conger may represent an excellent model for the study of reproduction in Anguilliformes.

## Introduction

The unique catadromous nature of eels (*Anguilla japonica, A. anguilla and A. rostrata*), make them a representative species of the phylogenetically ancient group of Teleostei [1], and because of this feature they are commonly used experimental animals for zoological studies that involve migration, environmental adaptation, and reproduction. This species grows in freshwater or in coastal areas of East Asia, but spawns offshore of the western North Pacific after extreme long-distance migrations [1–4]. Thus, eels captured in freshwater or inshore possess only immature gonads and naturally maturing fishes are not easily available. Furthermore, the gonads of eels remain dormant in culture conditions [5, 6]. Artificial gonadal development/maturation in cultured eels is partially possible by administration of gonadotropic hormone reagents, such as salmon pituitary extracts [7, 6, 8]. However, reports show that there are physiological and morphological differences between wild and artificially matured eels [4]. Therefore, it is imperative to study the reproductive biology, especially the process of natural gonadal maturation, of eels.

The daggertooth pike conger (*Muraenesox cinereus*; order Anguilliformes, family Muraenesocidae) is widely distributed in the Indo-West Pacific Ocean [9, 10]. Since pike conger is evolutionally primitive among Anguilliformes [10] and can be continuously captured by bottom trawling from the Seto Inland Sea, Japan [11], this fish may represent a useful model for studying Anguilliformes reproduction. However, such reports on the reproduction of daggertooth pike conger are completely lacking. Therefore, we took up this study with an aim to obtain basic information on the reproduction of pike conger.

As a first step, we examined the sex ratio, body size, age and gonadal maturity during the possible spawning season (July–October). Additionally, we analyzed the concentrations of sex steroid hormones (Estradiol-17ß and 11-keto testosterone, which are known to be involved in eel gametogenesis [12]), in plasma during oogenesis by enzyme-linked immunosorbent assay (ELISA). To our knowledge, this is the first report on the oogenesis of Anguilliformes in the wild.

## Materials and methods

### Animal

Wild daggertooth pike congers used for this study (*n* = 70) were collected from July to October 2013 by a commercial fisherman (Table 1). All samples were captured by trawl fishing from inshore waters (10–20 m depth) of Bisan strait, Okayama prefecture, Seto Inland Sea, Japan [34° 36′N, 134° 9′E].

**Table 1:** Daggertooth pike conger samples used in this study

Sampling date	Female		Male		Seawater tempreture^b^
	*N*	Body weight^a^	*N*	Body weight	
10-Jun-13	13	622.9 ± 142.6	0		20.1 °C
25-Jun-13	10	565.0 ± 110.8	0		20.9 °C
9-Jul-13	10	818.4 ± 77.13	0		24.0 °C
25-Jul-13	10	846.5 ± 281.0	1	337.6	24.4 °C
20-Aug-13	12	1149 ± 278.3	0		27.9 °C
18-Oct-13	12	752.6 ± 242.1	2	236.4	23.1 °C
Total	67		3		

^a^Body weight is mean ± SEM

^b^Tempreture is daily average

### Sampling procedures

After anesthesia, body weight and length were measured. Blood samples were collected from the caudal vein with heparinized syringes. After centrifugation, plasma was collected and stored at −30 °C until analysis. Eels were sacrificed by decapitation, and gonads and otoliths were removed. Gonads were weighed to calculate the gonadosomatic index (GSI = gonad weight/body weight × 100). Pieces of the gonad were fixed with Bouin’s solution for histological analysis. Otoliths were cleaned and stored dry until further analysis. All procedures were performed in accordance with the Guidelines for Animal Experimentation established by Okayama University.

### Age determination

To determine the ages of the individual fish, all otoliths were analyzed according to previously published method [13]. In brief, otoliths were embedded in polyester resin and then cut into 0.3 mm transverse sections with a saw microtome (SP1600, Leica Microsystems GmbH, Wetzlar, Germany). The sections were mounted on glass slides and their surfaces were ground to sequentially finer grades using carborundum paper. Finally, the sections were etched with 0.2 N HCl for 30 s. The number of rings (opaque zones) in the section, representing the age of individual fish, were counted under a microscope.

### Gonadal histology

To identify the sex and gonadal maturity, the fixed gonads were dehydrated and embedded in paraffin. Paraffin samples were serially sectioned to 7 μm and stained with hematoxylin and eosin. The determination of oocyte maturity was based on a previous report on common Japanese conger [14].

### Measurement of steroid hormones

The concentrations of estradiol-17β (E2) and 11-keto-testosterone (11KT), the major fish estrogen and androgen respectively, in the plasma were determined by enzyme-linked immunosorbent assay (ELISA). The analyses were performed according to a previous report [15], and absorbance was measured in a microplate reader (MTP-300; CORONA Electric Co. Ltd., Japan).

### Statistical analysis

Data on the steroid hormone levels and GSI are presented as means ± SEM. Significant differences were evaluated by one-way ANOVA, followed by Tukey–Kramer Multiple comparison test using PRISM 5.0b software (GraphPad, San Diego, CA). *P* < 0.05 was taken as the threshold for statistical significance.

## Results

### Sex ratio, age and growth

**Fig. 1:**
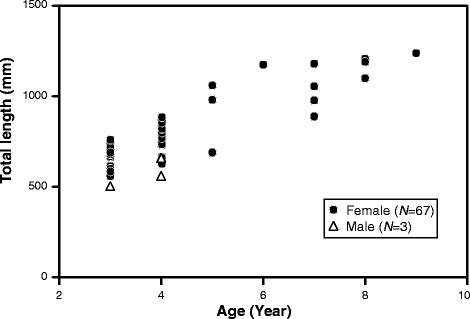
Age and total body length of daggertooth pike conger (*n* = 70). Closed circles indicate females and triangles indicate males

A total of 70 pike congers captured in inshore were used in this study. The sex of the samples was determined by histological observation. In our samples, females (*n* = 67) predominated over males (*n* = 3) (Table 1). The relationship between total length and age is shown in Fig. 1. The total lengths and ages ranged from 510 to 1239 mm and 3–9 years old, respectively. Males appeared to be smaller than females in length.

### Changes in gonadosomatic index in females

**Fig. 2:**
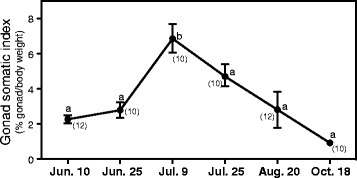
Changes in gonadosomatic index (GSI) in daggertooth pike conger females. Data are expressed as means ± SEM. Different alphabets denote statistical significance as analyzed by Tukey–Kramer multiple comparison test. Numbers in parentheses represent sample size

Changes in GSI in females during sampling period are shown in Fig. 2. GSI was 2.26 ± 0.77 at the first sampling (June 10), but rapidly increased to a peak by 9 July (6.86 ± 2.58). Thereafter, the GSI decreased gradually, reaching a minimum by 18 October (0.98 ± 0.31).

### Histological observation of the ovary

**Fig. 3:**
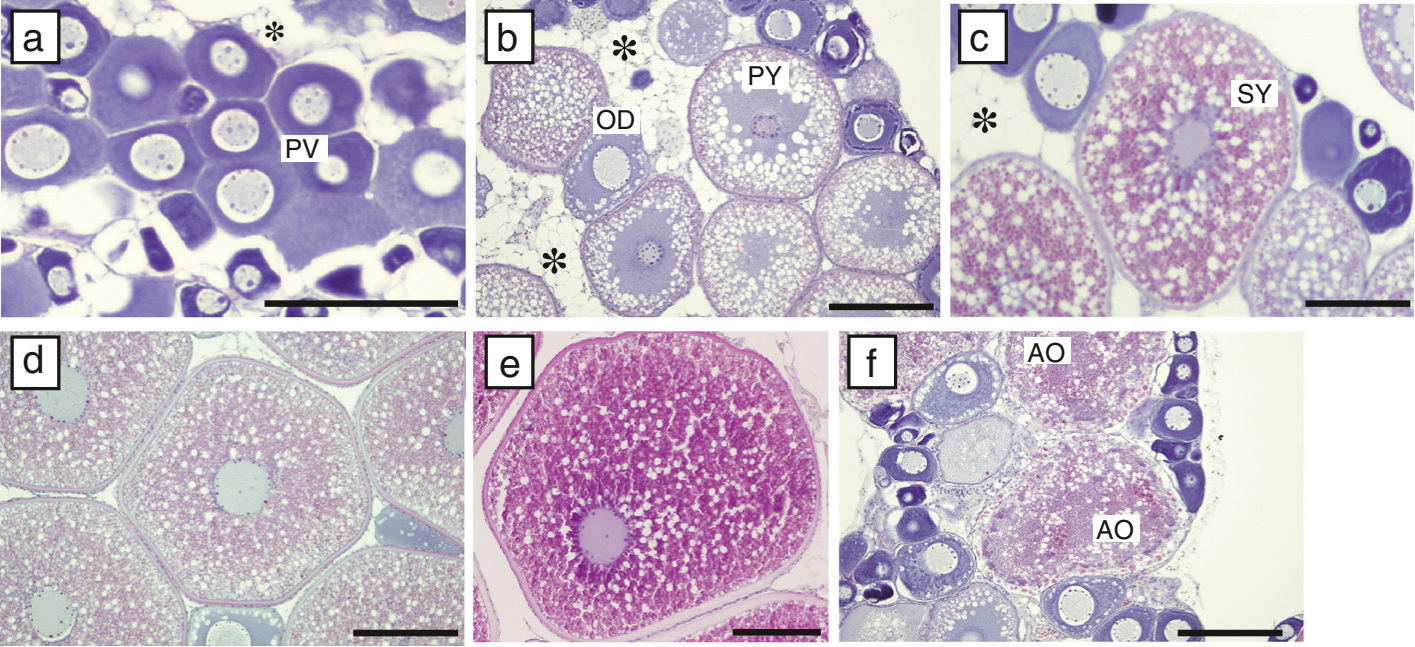
Photomicrographs of ovaries in wild daggertooth pike conger. (**a**) Stage I: immature. (**b**) Stage II: vitellogenesis onset. (**c**) Stage III: vitellogenesis progression. (**d**, **e**) Stage IV: vitellogenesis completion. (**f**) Stage V: post-spawning. Asterisks (*) indicate adipose cells. Bars: 200 μm. Abbreviations: PV, pre-vitellogenic oocytes; OD, oil droplet stage oocytes; PY, primary yolk stage oocytes; SY, secondary yolk stage oocytes; AO, atretic oocytes

Gonadal histological observation revealed that the ovary of pike conger contained oocytes at various developmental stages (Fig. 3). In addition, adipose cells were observed in the ovaries of all samples (Fig. 3).

Based on the most advanced maturity of oocyte in the ovary, we classified the individual females into five stages (Table 2 and Fig. 3).

**Table 2:** Maturity stages of ovary in the daggertooth pike conger

Stage		Oocyte stage (oocyte diameter)	Figure 3
I	Immature	Pre-nucleous (<130 μm)	A
II	Vitellogenesis onset	Oil droplet stage (130–180 μm)	B
		Primary yolk stage (200–330 μm)	
III	Vitellogenesis progression	Secondry yolk stage (320–450 μm)	C
IV	Vitellogenesis completion	Tertiary yolk stage (500–800 μm)	D
		Migratory nucleus stage (700–900 μm)	E
V	Post-spawaning	Atretic oocyte	E

#### Stage I

Immature. Fish at this stage had ovaries containing many pre-vitellogenic oocytes. No mature oocytes were observed in the ovary (Fig. 3a).

#### Stage II

Vitellogenesis onset. The ovary of this stage harbored oil droplets and primary yolk oocytes (Fig. 3b).

#### Stage III

Vitellogenesis progression. Many oocytes at the secondary vitellogenic stage were observed in the ovary (Fig. 3c).

#### Stage IV

Vitellogenesis completion. Many tertiary yolk-stage oocytes (Fig. 3d) and few migratory nucleus-stage oocytes (Fig. 3e) were observed in the ovary. This stage was observed in the youngest sample also (age 3).

#### Stage V

Post-spawning. The ovaries of this stage contained atretic oocytes (Fig. 3f).

### Changes in female maturation

**Fig. 4:**
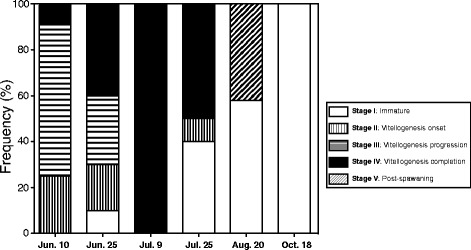
Changes in female maturation during sampling period

Changes in female maturation during the sampling period are shown in Fig. 4. Ovaries of fish captured in June showed active vitellogenesis (stage II and III). All females of 9 July were in stage IV. Fishes in the stage V appeared in August. All fishes of October were stage I.

### Plasma steroids in female

**Fig. 5:**
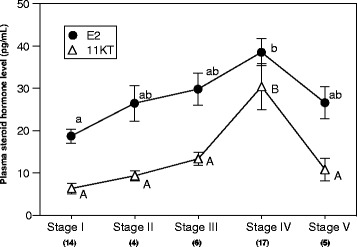
Plasma estradiol-17β (E2) and 11-keto-testosterone (11KT) levels during female maturation. Data are expressed as means ± SEM. Closed circles indicate E2 level and triangles indicate 11KT level. Different alphabets denote statistical significance. Numbers in parentheses represent sample size

The plasma levels of sex steroid hormones during female maturation are shown in Fig. 5. The levels of E2 were maintained throughout different maturity stages, with peak in stage IV (Fig. 5). The level of 11KT was significantly higher in stage IV than that of other stages (Fig. 5).

### Histological observation of testis

**Fig. 6:**
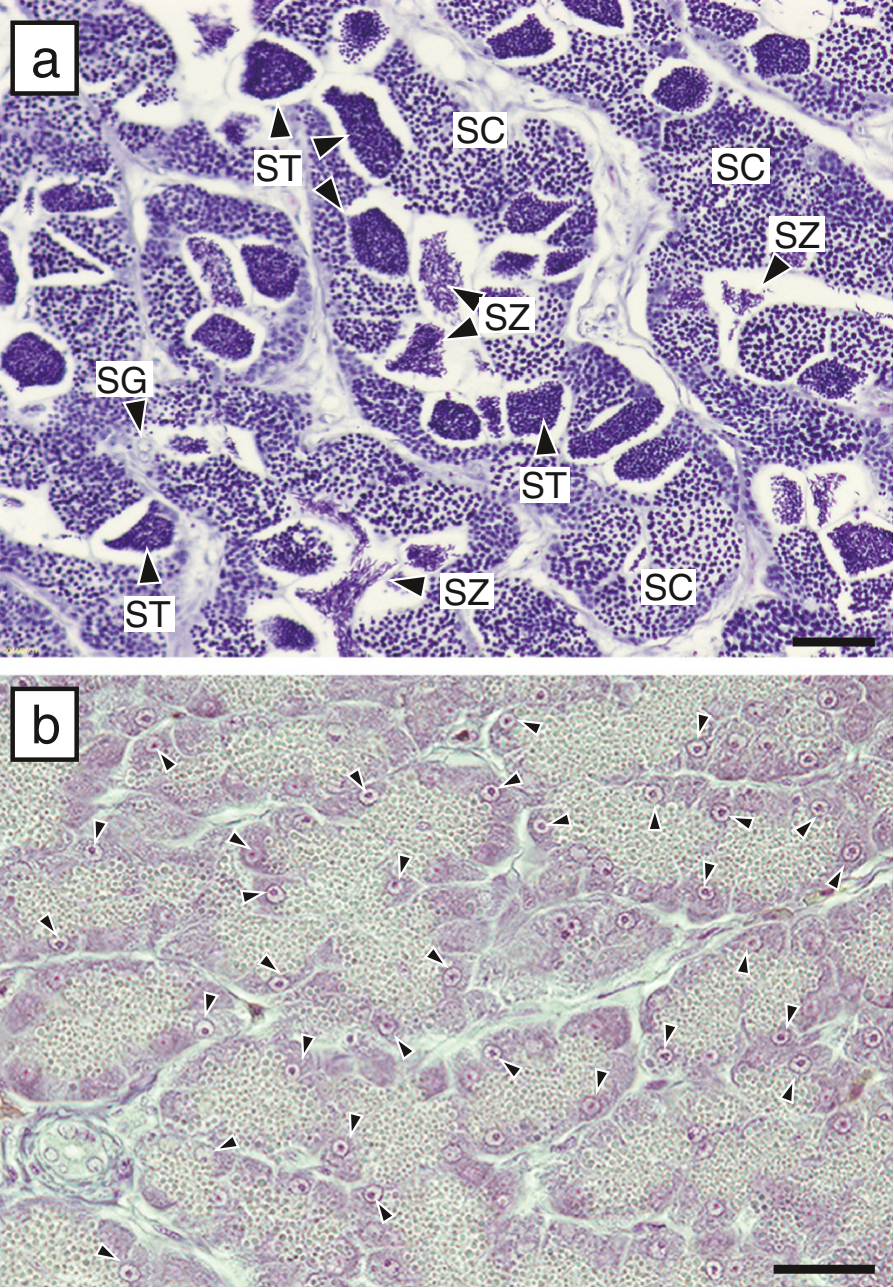
Photomicrographs of testes in daggertooth pike conger. (**a**) Male with all stages of spermatogenesis (25 July, GSI, 2.07 %; TL, 665 mm) and (**b**) male with only spermatogonia (arrowheads) (18 October, GSI, 0.079 %; TL, 510 mm). Bars: 50 μm. Abbreviations: SG, spermatogonia; SC, spermatocyte; ST, spermatid; SZ, spermatozoon

Few males (*n* = 3) were captured in this study. One male captured on 25 June had mature testis showing various stages of spermatogenesis, including sperm (Fig. 6a). In contrast, two males (October) had immature testes comprising only spermatogonia with adipose cells (Fig. 6b).

## Discussion

In this study, we investigated the basic reproductive biology of daggertooth pike conger. First, we examined the sex ratio, age and body size. Second, the oogenesis of pike conger was characterized by the histological observation and the spawning period was determined.

The changes in GSI and maturity indicate that the pike conger’s spawning season is around July. Gonadal histological observation revealed that pike conger has an asynchronous type of ovary, containing oocytes at various developmental stages, and that this fish is mature by at least 3-years-old, which is the youngest sample in this study. Considering the general rule that fish with the asynchronous type of ovary spawn several times during one spawning season [16], pike conger at least 3-years-old could spawn several times around July.

We found that the sex ratio was skewed towards females. Although the reason for this fact could not be established in this study, similar sex ratio imbalances have also been reported in other Anguilliformes [17–19]. In the case of the wild European conger eel (*Conger conger*), females were captured exclusively in the shallow coastal waters, whereas males were observed in deeper waters [17]. In a recent study on the wild Japanese eel, large variations in sex ratio were observed among the sampling locations [18]. Considering these previous reports and the present study, it appears that the habitats of Anguilliformes females and males may differ in the wild, although this needs to be confirmed by capturing pike congers from different sites or by bio-logging of pike conger females and males.

As described in the Introduction, *A. anguilla, A. rostrata and A. japonica* captured inshore always have immature gonads, and their spawning areas have been reported to be located offshore in the open ocean [20, 1], making it difficult to use these fish for studies of eel reproduction. In contrast, pike congers captured inshore have matured testes and ovaries with adipose cells, which are the typical features of Anguilliformes [17, 21–24]. Therefore, pike conger may represent a better model for studying the reproduction of Anguilliformes.

Histological observation also revealed that the migratory nucleus stage oocytes were present in the ovary of pike conger (stage IV). It is well established that this stage oocytes immediately precede hydration and ovulation [25, 26]. The spawning area of pike congers thus appears to be close to our sampling area in the Seto Inland Sea, although the precise location remains unknown. Unlike in catadromous Anguilliform species [20], changes in the external morphological features for spawning migration, such as skin color, eye-size, and color of the pectoral fin [27], have not been observed in the pike conger.

In this study, we measured the concentrations of sex steroid hormones (E2 and 11KT) in plasma during oogenesis (Fig. 5). However, the concentrations of both hormone were lower than that of other eel species (average concentration of E2 and 11KT in pike conger are 28.4 and 17.5 ng/ml, respectively) [23, 28]. These results raise the possibility that major steroid hormones of pike conger are different from other eel species.

Although the plasma level of E2 is low in pike conger, changes of this hormone during oogenesis were largely consistent with artificially matured Japanese eel induced by salmon pituitary extracts [29]. Thus, E2 play important roles in oocyte growth (vitellogenesis) in pike conger, as well as Japanese eel.

Level of 11KT in pike conger increased gradually during vitellogenesis, similar to other eel species [29]. In Japanese eel, 11KT is thought to be involved for control of lipid droplet accumulation into oocytes [30]. In addition, ability of androgen to induce the spawning migration and silvering was reported in some eels [31–33]. Therefore, 11KT may play an important role in vitellogenesis and spawning migration in pike conger.

## Conclusions

In this manuscript, we report that daggertooth pike conger in the Seto Inland Sea have mature gonads. Because naturally maturing fish can easily be captured and induced to spawn in captivity without any hormone treatment [34], this fish should be considered a potentially useful model for studies of reproduction in Anguilliformes. The importance of such research is highlighted by the depletion in natural stocks of some eel species in recent years, making them critically endangered [35]. In this context, novel approaches to restore the natural resources of eels (e.g., the observation of the spawning behavior, test of new artificial maturation-inducing reagents, *in vivo* transplants of germ cells isolated from endangered eel species, and so on) can possibly be explored using pike conger as a valuable model.
